# Real-world treatment patterns of renal anemia in hemodialysis patients: A multicenter cohort study performed using DialysisNet (RRAHD study)

**DOI:** 10.1097/MD.0000000000018749

**Published:** 2020-01-10

**Authors:** Hyo Jin Kim, Ji In Park, Kyung Don Yoo, Yunmi Kim, Hyunjeong Baek, Sung Ho Kim, Taehoon Chang, Hye Hyeon Kim, Kye Hwa Lee, Seungsik Hwang, Clara Tammy Kim, Hoseok Koo, Ju Han Kim

**Affiliations:** a Department of Internal Medicine, Pusan National University Hospital, Busan; b Department of Internal Medicine, Kangwon National University School of Medicine; c Department of Internal Medicine, Kangwon National University Hospital; d Division of Nephrology, Department of Internal Medicine, Ulsan University Hospital, Ulsan; e Department of Internal Medicine, Inje University Busan Paik Hospital; f Seoul National University Biomedical Informatics (SNUBI), Division of Biomedical Informatics, Seoul National University College of Medicine; g Department of Public Health Science, Graduate School of Public Health, Seoul National University; h Institute of Life and Death Studies, Hallym University, Chuncheon; i Department of Internal Medicine, Seoul Paik Hospital, Inje University College of Medicine; j Systems Biomedical Informatics Research Center, Seoul National University; k Center for Precision Medicine, Seoul National University Hospital, Seoul, Republic of Korea.

**Keywords:** anemia, dialysis, hemodialysis, real-world, treatment

## Abstract

A multicenter cohort study.

The DialysisNet was previously developed for the management of hemodialysis (HD) patients based on the American Society for Testing and Materials Continuity of Care Records by metadata transformation. DialysisNet is a dialysis patient management program created by using the personal health record care platform to overcome the problems of registry studies, in real-time.

Here, we aimed to investigate the pattern of treatment for renal anemia in HD patients using DialysisNet.

We performed a multicenter cohort study among HD patients who were treated at one of the three Korean university-affiliated hospitals from January 2016 to December 2016. Subjects were divided into 4 hemoglobin variability groups by quartiles. The variable anemia treatment pattern was reviewed. To determine renal anemia treatment patterns, we automatically collected information on the practice of anemia treatment patterns such as erythropoietin stimulating agent (ESA) doses and administration frequencies, and targeted hemoglobin maintenance rate. Individual hemoglobin variabilities were expressed as (standard deviations)/(√(n/[n–1]).

The records of 159 patients were analyzed (Hospital A: 35, Hospital B: 21, Hospital C: 103). Mean patients’ age was 65.6 ± 12.8 years, and 61.6% were men. Overall, hemoglobin level was 10.5[7.43;13.93] g/dL. 158 (99.3%) patients were using ESA; and overall, the epoetin alfa dose was 33,000[4000;136,800] U per week. Hemoglobin levels (*P* = .206) and epoetin alfa doses were similar (*P* = .924) for patients with different hemoglobin variabilities. The hemoglobin target maintenance rate was lower in the highest hemoglobin variability group than in the lowest variability group (*P* = .045).

In this study, detailed information on the actual anemia treatment patterns were obtained using the DialysisNet. We expect that DialysisNet will simplify and improve the renal anemia management for both dialysis patients and health care providers.

## 1 Introduction

Anemia is common in hemodialysis (HD) patients; it usually develops as a result of erythropoietin deficiency, and leads to considerable morbidity and mortality.^[1]^ Optimal management of anemia in HD patients is associated with improved quality of life and reduced hospitalization and death.^[2–4]^ Anemia management was improved by the introduction of erythropoietin stimulating agent (ESA) in 1989, which reduced blood transfusions and iron overload complications. As a result, ESA is now considered a well-tolerated and effective treatment, and its clinical benefits in HD patients have been well reported.^[5,6]^ However, recent studies have shown that higher doses of ESA administered for the treatment of anemia are associated with poor outcomes.^[7–9]^ Koulouridis et al^[10]^ showed by meta regression analysis that higher ESA doses might be associated with increased all-cause mortality and cardiovascular complications independently of hemoglobin level. Since lower ESA doses might prove to be beneficial to our patients, closer surveillance of both ESA doses and hematocrit/hemoglobin levels could be suggested.

Few reports have been issued on dialysis patients in Korea. The Korea Society of Nephrology (KSN) registration process is limited because laboratory results are obtained at the time of initial registration and are not well updated, and also the recent treatment behavior could not be reflected.

The Dialysis Outcomes and Practice Patterns Study (DOPPS) and the United States Renal Data System (USRDS) are 2 representative renal studies. DOPPS is a leading source of up-to-date, representative, and comprehensive data on HD practice and patient outcomes worldwide,^[11]^ while USRDS maintains an extensive database that facilitates biomedical and economic research on end-stage renal disease (ESRD) in the US population.^[12]^ However, the type of data recorded is limited, and is not reflected on a real-time basis, and data expansion is difficult. In the case of DOPPS, patients are chosen at random,^[13,14]^ and much manpower is required to register dialysis patients.^[14]^

To generalize and cross validate ideas, physicians require data from larger numbers of cases, but research based on hospital electronic medical record (EMR) systems is not possible because of the different systems used in different hospitals used. For this reason, a gap exists between available medical information and data suitable for analytical purposes, resolving this requires much manpower and expense. To overcome these problems, we created the Health Avatar Care Platform, which was designed for the management of chronic diseases. It is wholly compatible with the ISO/IEC 11179 based system. Clinical data could be exchanged on iPad/Smartphone using this platform.^[15]^ DialysisNet is a dialysis patient management program created using the Health Avatar Care Platform.

DialysisNet is a dialysis center management program for doctors, based on the Continuity of Care Record + (CCR +), and is currently used at 3 university-affiliated hospitals in Korea (Seoul Paik Hospital, Dongguk University Gyeongju Hospital, and Kangwon National University Hospital). Furthermore, it can be used to check the anemia status of dialysis patients using average hemoglobin results with variables defined in DOPPS. DialysisNet also allows doctors to view prescriptions related to anemia,^[15,16]^ and enables records of real-time medical treatment to be converted into research results. Currently, data from individual hospitals is transmitted to BioEMR servers in an anonymized manner, which allows results to be analyzed and viewed in real-time.^[17]^

Using DialysisNet, the BioEMR, and the CCR + a standard, it is possible to examine anemia management in HD patients in real-time, and to prevent errors associated with registration and data processing. Therefore, in the present study, we aimed to evaluate real-world treatment pattern of renal anemia in HD patients (RRAHD study) using DialysisNet which requires less man power and were less errors.

## 2 Methods

### 2.1 Common data modeling of anemia for the multicenter trial

We revised the common data elements configured for the CCR of HD, which was previously constructed^[15]^ to be compatible with that of the multicenter anemia studies on HD patients. The common data elements used in the present multicenter trial included demographics, diagnoses, prescription drugs, dialysis information, and laboratory results. These common data elements were separated into content lists for demographics (12 items), diagnoses (7 items), prescription drug (14 items), dialysis information (43 items), and laboratory (55 items).We defined and collected anemia-related variables in a common data model. Table 1 shows the common data elements used in the multicenter anemia studies.

**Table 1:**
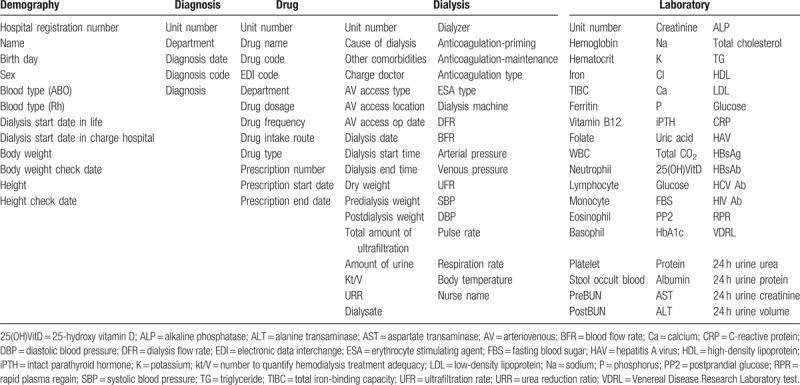
Common data elements used in the multicenter trial.

### 2.2 Compliance method with standard models and development of a multicenter trial concept in DialysisNet

To extend the common data model to DialysisNet for multicenter studies, common data model was converted to the American Society for Testing and Materials Continuity of Care Record (ASTM-CCR)^[18]^ standard for the representation of medical information.^[15]^ For the compliance method, ISO/IEC 11179 (the standard for data transmission) was used. The common data model was represented in the metadata via ISO/IEC 11179 and verified using the ASTM-CCR/Extensible Markup Language (XML) schema definition XML Schema Definition (XSD) before being parsed into the database. It was stored in the CarePlatform using the representational state transfer (REST) protocol.^[15]^ This data was then encrypted and sent to BioEMR for collaborative research.

### 2.3 Development of BioEMR for multicenter trials in DialysisNet

The BioEMR is a system that provides clinical and bioinformation integration and is equipped with sharing technology.^[17]^ BioEMR consists of the following: an extract or that which converts hospital information and clinical genetic information into XML; an Integrated Development Environment (IDE), which is a repository of XML; integrated analysis part for IDE data; case report form; part of common data element converted to Clinico-Histopathological Metadata Registry (CHMR); and the verifying part of the terminology. By using standard terminologies, we created metadata and operate/reposite forms. Clinical documents were also received in XML format, and after metadata validation, stored in the clinical results database.

In each hospital EMR system, order communication system (OCS) data are converted using the Health Level Seven (HL-7) standard^[19]^ while laboratory information system data are converted using the Logical Observation Identifiers Names and Codes (LOINC) standard.^[20]^ After XML representation is performed through the BioEMR extractor, it is stored in the CHMR format, and by metadata-validation enhancement data indexing, is gathered into research data.

### 2.4 Study population

We performed this multi-center, retrospective study at 3 Korean university-affiliated hospitals (Seoul Paik Hospital, Dongguk University Gyeongju Hospital, and Kangwon National University Hospital). The data of patients ≥18 years of age, who received HD >twice per week and >8 times per month for >3 months, from January 2016 to December 2016 at these 3 university-affiliated hospitals, were analyzed. This study was approved by the Institutional Review Boards at Seoul Paik Hospital (IIT-2016–216), Dongguk University Gyeongju Hospital (110757–201608-HR-06–01), and Kangwon National University Hospital (KNUH-2016-08-006). The requirement for patient informed consent was waived because of the retrospective nature of the study. All clinical investigations were conducted in accordance with the principles of the Declaration of Helsinki.

### 2.5 Clinical data collection and laboratory evaluations

Data related to anemia over 1 year was analyzed. Values closest to the first day of each month were used. Data were extracted from the common data model of DialysisNet. The anemia-associated variables analyzed were as follows: hemoglobin value, hemoglobin test date, hematocrit, hematocrit test date, iron/total iron-binding capacity (TIBC)/ferritin value, iron/TIBC/ferritin test date, ESA type, ESA dose, ESA prescription date, Kt/V, aspartate aminotransferase (AST) value, alanine aminotransferase (ALT) level, and angiotensin-converting enzyme inhibitor (ACEi), and angiotensin II receptor blocker (ARB) prescription. TSAT was calculated using; 100 × serum iron/TIBC (%).

### 2.6 Definitions used in this study

Target hemoglobin value: The target value was set using the average hemoglobin test results and ratios as defined by the anemia categories of DOPPS (hemoglobin < 10, 10–10.99, 11–11.99, ≥12 g/dL).^[4]^

Changes in the mean hemoglobin values, that is, intrapersonal means and standard deviations (SDs) of all measured hemoglobin values, were calculated for all subjects. Intrapersonal SDs of serially-measured hemoglobin values were adjusted for the numbers of assessments per subject. Hemoglobin level variability was defined as (SD of hemoglobin/(√[n/(n–1)]), and subjects were divided into 4 hemoglobin variability groups by quartile.

Two types of ESA, epoetin alfa and darbepoetin alfa, were prescribed. ESA dose was standardized to weekly epoetin alfa equivalent. Darbepoetin alfa and epoetin alfa doses were converted using the ratio 1:300 (μg darbepoetin alfa to international units of epoetin alfa).^[21]^

As an operating system for the treatment of dialysis, DialysisNet enables access to extensive dialysis-related data. DialysisNet is a physician's tool, which simplifies the dialysis center data management based on the standard CCR personal health records. The dialysis center management component of DialysisNet was based on DOPPS items.

### 2.7 Statistical analyses

The analysis was performed using R version 3.1.1. (R Foundation for Statistical Computing, Vienna, Austria). Results are presented as means ± SDs for normally distributed variables and as median [interquartile range] for variables with a skewed distribution. Categorical variables presented as frequencies and percentages. For continuous variables, to determine patient characteristics, descriptive analysis was performed. Categorical variables were analyzed using the Chi-square test to determine the natures of correlations between variables. We allocated subjects to 4 groups by hemoglobin variability level quartile for comparative analysis. Analysis of variance or the Kruskal–Wallis test were used to determine the significances of intergroup differences. *P-*values of <.05 were considered statistically significant.

## 3 Results

### 3.1 Demographic and clinical characteristics of subjects

The clinical characteristics of the study subjects are shown in Table 2. We analyzed 159 patient records (Hospital A: 35, Hospital B: 21, and Hospital C: 103) using common data elements for HD information including anemia-associated attributes. Mean overall subject age was 65.6 ± 12.8 years and 61.6% were men. Patients with diabetes mellitus (DM) and hypertension comprised 52.2% and 88.0% of the subjects, respectively. Mean dialysis vintage was 62.6 ± 58.0 months and mean Kt/V was 1.61 ± 0.54. Albumin level was 3.8[2.4;4.5] g/dL and no difference was observed between hospitals.

**Table 2:**
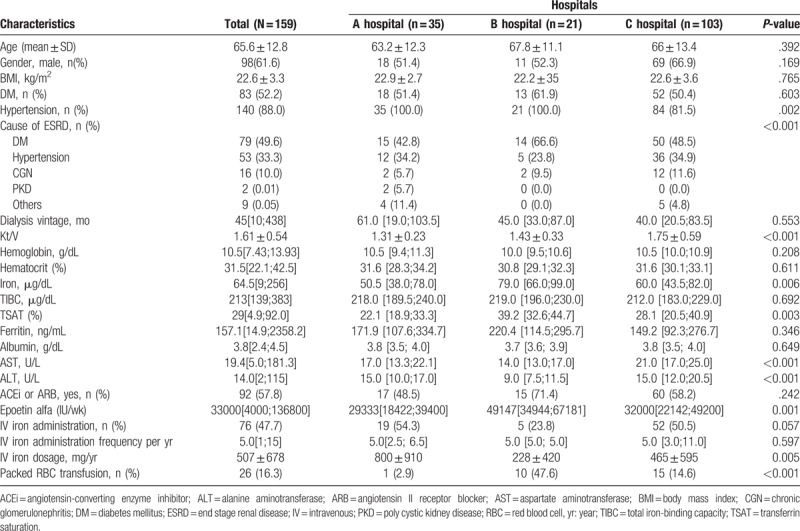
Clinical characteristics of the study subjects at the 3 hospitals.

### 3.2 Anemia-related parameters and doses of epoetin alfa at participating hospitals

**Figure 1:**
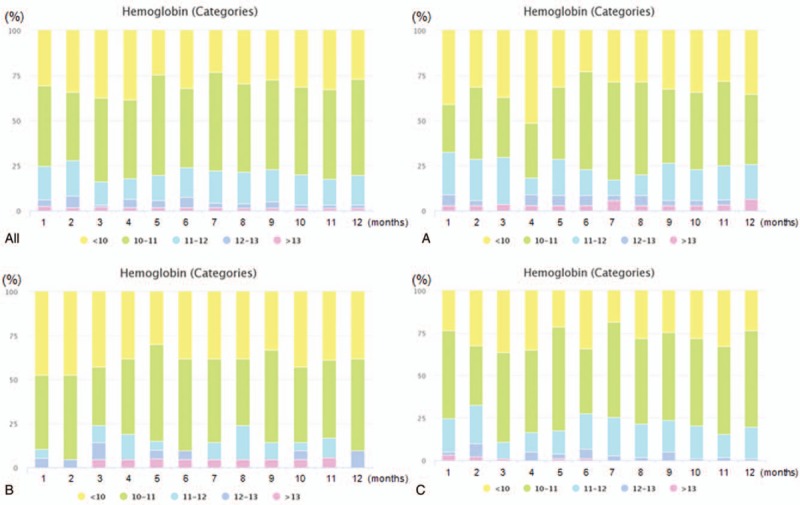
Distribution of hemoglobin level by hospital. The percentage of hemoglobin categories accounted for about 50% and 20% of 10 to 11, and 11 to 12 g/dL hemoglobin level, respectively. (all): all hospitals, (A): Hospital A, (B): Hospital B, (C): Hospital C.

**Figure 2:**
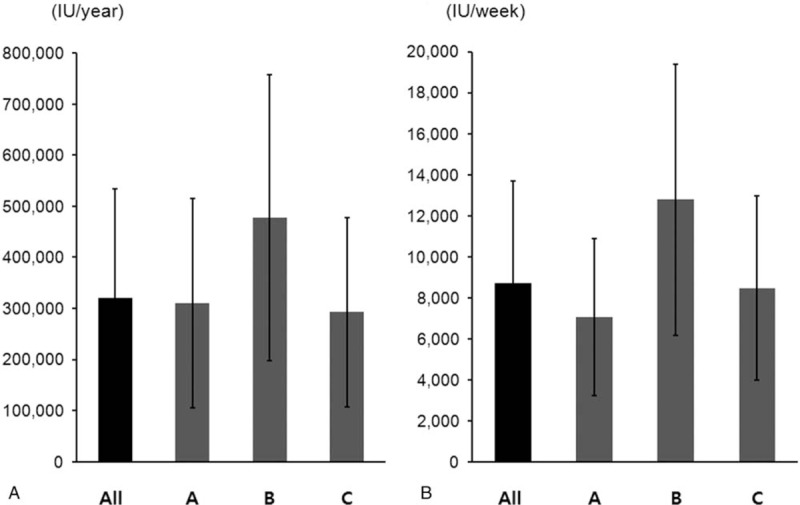
Mean doses of epoetin alfa at the 3 participating hospitals. (A) Mean epoetin alfa dose per annum at the 3 hospitals. Mean annual usage of epoetin alfa was 321,308 ± 212,388 U per patient (Hospital A: 311,063 ± 204,676 U/yr, Hospital B: 477,714 ± 279,531 U/yr, Hospital C: 292,601 ± 185,992 U/yr). (B) Mean overall weekly dose of epoetin alfa per week was 8731 ± 4947 U (Hospital A: 7052 ± 3825 U/wk, Hospital B: 12,798 ± 6,615 U/wk, Hospital C: 8462 ± 4489 U/wk).

Overall hemoglobin level was 10.5[7.43;13.93] g/dL (Hospital A: 10.5 [9.4;11.3] g/dL, Hospital B: 10.0 [9.5;10.6] g/dL, and Hospital C: 10.5 [10.0;10.9] g/dL; *P* = .343). Figure 1 shows the hemoglobin categorical graph view of all hospitals. BioEMR enabled us to examine the monthly hemoglobin levels by DOPPS standard category. The percentage of hemoglobin category accounted for about 50% and 20% of 10 to 11 and 11 to 12 g/dL hemoglobin levels, respectively. Overall TSAT (transferrin saturation) was 29[4.9;92.0]% (Hospital A: 22.1 [18.9;33.3]%, Hospital B: 39.2 [32.6;44.7]%, and Hospital C: 28.1 [20.5;40.9]%; *P* = .003; Table 2). Mean overall ferritin level was 157.1[14.9;2358.2] ng/mL (Hospital A: 171.9 [107.6;334.7] ng/mL, Hospital B: 220.4 [114.5;295.7] ng/mL, and Hospital C: 149.2 [92.3;276.7] ng/mL; *P* = .346). The mean dose of iv iron was different among hospitals (Hospital A: 800 ± 910, Hospital B: 228 ± 420, and Hospital C: 465 ± 595; *P* = .005). The transfusion of RBC was different among hospitals (Hospital A: 1(2.9), Hospital B: 10(47.6), and Hospital C: 15(14.6); *P* < .001). The Overall, 158 (99.3%) patients were using ESA. Patients in Hospital B were administered with larger amount of ESA doses (Fig. 2). Mean overall epoetin alfa dosage per annum was 321,308 ± 212,388 U (Hospital A: 311,063 ± 204,676 U/yr, Hospital B: 477,714 ± 27,9531 U/yr, and Hospital C: 292,601 ± 185,992 U/yr; Fig. 2A). Mean overall dose of epoetin alfa per week was 8731 ± 4947 U (Hospital A: 7052 ± 3825 U/wk, Hospital B: 12,798 ± 6615 U/wk, and Hospital C: 8462 ± 4489 U/wk; Fig. 2B).

### 3.3 Relationships between hemoglobin variability grade, hemoglobin level, and epoetin alfa dose

**Figure 3:**
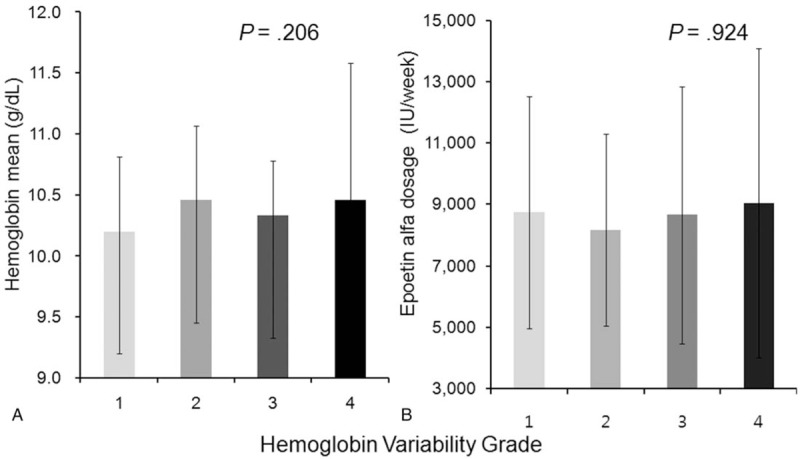
Relationships between hemoglobin variability grades, hemoglobin level, and mean epoetin alfa dose. (A) Hemoglobin levels (treated as a continuous variable) were independent of hemoglobin variability grade (*P* = .206). (B) Similarly, epoetin alfa doses were similar for different hemoglobin variability grades (*P* = .924).

**Figure 4:**
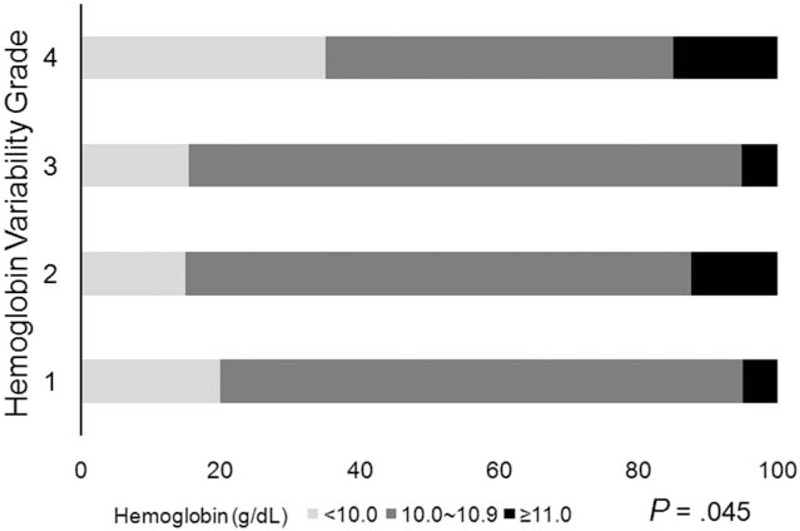
Relationship between hemoglobin level and hemoglobin variability grade. The proportion of patients with hemoglobin level of <10 g/dL was greatest among those with hemoglobin variability grade 4. The hemoglobin maintenance rate was lower in the higher variability group 4 than in the other groups (*P* = .045).

Hemoglobin level variability was defined as (SD of hemoglobin/(√[n/(n–1)]). No significant relationship was found between hemoglobin (treated as a continuous variable) (Fig. 3A, *P* = .206) or epoetin alfa dose, and annual basis hemoglobin variability grade (Fig. 3B, *P* = .924). The proportion of patients with hemoglobin level of <10 g/dL was greatest among those with hemoglobin variability grade 4 (Fig. 4). The hemoglobin maintenance rate was lower in the higher variability group 4 than in the other groups (*P* = .045). In multiple logistic regression analysis, after adjusting for Kt/V, IV iron dosage, transfusion frequency and epokine dose, the hemoglobin variability significantly affected the prevalence of anemia (not normal range 10.0–11.0) (odds ratio [OR] 5.77; 95% confidence interval, 1.381–26.109, *P* = .018) (Table 3).

**Table 3:**
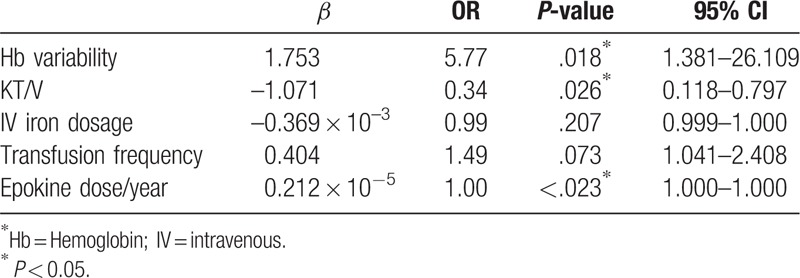
Multiple logistic regression to determine the factors identifying for anemia (not normal range 10.0–11.0).

## 4 Discussion

In the present study, we evaluated the real-world anemia management in Korea using a novel tool, DialysisNet, devised by our group, which requires less man power and were less errors. By analyzing accumulated data in a standardized format, we were readily able to compare hemoglobin levels and ESA doses at 3 different centers. Patients at these 3 centers showed similar baseline characteristics mostly, including hemoglobin level, although ESA doses differed. Interestingly, higher hemoglobin variability group was found to be significantly associated with a lower hemoglobin maintenance rate (10–10.9 g/dL).

In this study, the mean overall hemoglobin level during the study period was 10.5 (7.4, 13.9) g/dL, and ESA was administered to 99.3% of the study subjects. According to KSN registry results for 2015, mean hemoglobin level of HD patients was 10.5 ± 1.17 g/dL, and 85% of patients had used ESA.^[22]^ In the nationwide prospective cohort, Korean Clinical Research Center for End-Stage Renal Disease Study (CRC-ESRD), 71.8% of HD maintenance patients received ESA at a mean dose of 8858.5 U per week.^[23]^ Our results regarding the mean hemoglobin level and ESA dose concurred with those previously reported using representative Korean data, although the proportion of those that received ESA was higher. The reason for the discrepancy is that our data reflect the real clinical practice management. The KSN registry and the CRC-ESRD study analyzed data cross-sectionally; thus, 15% to 28% of patients were not using ESA at the time of sampling, whereas in practice, 99.3% of patient were prescribed with ESA on an annual basis. When our results were compared with those obtained in other countries, mean hemoglobin level determined in the present study (10.3 ± 1.1 g/dL) was comparable with those reported in Japan and United States (both 10.6 g/dL) and lower than that reported in Europe (11.2 g/dL based on the latest DOPPS 5 data).^[24]^ DOPPS 5 also reported that the median weekly ESA dose by region ranged from 5000 (Japan) to 8655 U (United States); the mean weekly ESA dose in the present study was 8731 U.

Hospital B was found to use larger amount of ESA doses than the other 2 centers, which we attributed to a significantly higher proportion of diabetic ESRD patients and older age, though not statistically significant. Diabetic kidney disease-related anemia developed earlier and was more severe compared with non-diabetic kidney disease-related anemia due to inflammatory cytokines, poor response to ESA, and so on.^[25]^ In addition, hematopoietic environment and nutritional status become poor in the elderly.^[26]^ This would have affected the use of larger amount of ESA doses at hospital B.

Despite similar ESA doses, the maintenance rate of hemoglobin at 10 to 10.9 g/dL was lower in hemoglobin variability group 4 than in the other 3 groups. The concept of hemoglobin variability in HD patients was first described in 2003 by Lacson et al^[27]^ and Berns et al.^[28]^ Yang et al^[29]^ concluded that greater hemoglobin variability is independently associated with higher mortality in HD patients. However, a subsequent study showed that after adjustment for disease severity, evidence supporting a link between intra-patient hemoglobin variability and mortality was weak and inconsistent.^[30]^ Nevertheless, though hemoglobin variability did not independently affect mortality in this previous study, we found those exhibiting lower variability maintained the target hemoglobin range on similar ESA doses. The percentage of those with a hemoglobin of <10 g/dL, which was proven to be a predictor of poor outcome, was also significantly higher in hemoglobin variability group 4. Thus, we suggest that physicians should try to reduce the variability, and maintain the target hemoglobin range. Kalantar-Zadeh and Aronoff^[31]^ proposed several useful strategies for reducing hemoglobin variability.

This is the first clinical study to utilize DialysisNet, which collects biodata of HD patients using a common data model. Although, we did not any intervention using DialysisNet, we showed real-world anemia management pattern using repeated biodata of HD patients easily. DialysisNet has several advantages in the research field. First, it simplifies data analysis and eliminates considerable efforts required to collect data on different variables from the EMR at different centers. Secondly, it enables real-time analysis. Currently, the Korean KSN ESRD registry collects nationwide data on dialysis using an on-line registry program, but it only collects data once a year and the response rate is about 67%.^[22]^ On the other hand, DialysisNet allows for serial data collection in real-time without the loss of data. Third, the risk of human errors is minimal, because data are sent to the DialysisNet server automatically from hospital EMRs.

The DialysisNet is being rapidly adopted in Korea and is now installed at 15 centers including 1 primary dialysis clinic, 8 secondary, and 6 tertiary hospitals. Though this first study on the system included only 3 centers, in the future it could provide nationwide coverage. Furthermore, because the routine management of HD is standardized internationally, it is hoped that in the near future it will be adopted by other countries.

Several limitations of the present study warrant consideration. In the analysis we calculated epoetin alfa-equivalent doses, which is an oversimplification since ESAs have different properties.

This study shows that by using DialysisNet, detailed information on real-world anemia treatment pattern can be readily accessed. We hope that DialysisNet will improve the management of renal anemia in dialysis patients and simplify the management for health care providers and patients.

## Author contributions

**Conceptualization:** Hyo Jin Kim, Ji In Park, Kyung Don Yoo, Hoseok Koo, Ju Han Kim.

**Data curation:** Hyo Jin Kim, Ji In Park, Kyung Don Yoo, Yunmi Kim, Hyunjeong Baek, Sung Ho Kim, Taehoon Chang, Hye Hyeon Kim, Kye Hwa Lee, Seung-sik Hwang, Hoseok Koo, Ju Han Kim.

**Formal analysis:** Hyo Jin Kim, Ji In Park, Kyung Don Yoo, Sung Ho Kim, Taehoon Chang, Hye Hyeon Kim, Hoseok Koo.

**Funding acquisition:** Ju Han Kim.

**Investigation:** Hyo Jin Kim, Ji In Park, Kyung Don Yoo, Yunmi Kim, Hyunjeong Baek.

**Methodology:** Kyung Don Yoo, Sung Ho Kim, Taehoon Chang, Hye Hyeon Kim, Kye Hwa Lee, Seung-sik Hwang, Ju Han Kim, Clara Tammy Kim.

**Software:** Sung Ho Kim, Taehoon Chang, Hye Hyeon Kim, Kye Hwa Lee, Ju Han Kim.

**Supervision:** Seung-sik Hwang, Hoseok Koo, Ju Han Kim.

**Validation:** Clara Tammy Kim.

**Writing – original draft:** Hyo Jin Kim, Ji In Park, Kyung Don Yoo.

**Writing – review & editing:** Hyo Jin Kim, Ji In Park, Hoseok Koo, Ju Han Kim.

Hyo Jin Kim orcid: 0000-0001-9289-9073.

Ji In Park orcid: 0000-0003-4662-3759.

Kyung Don orcid: Yoo 0000-0001-6545-6517.

Yunmi Kim orcid: 0000-0001-9281-9926.

Hyunjeong Baek orcid: 0000-0003-1325-3392.

Sung Ho Kim orcid: 0000-0003-1850-8251.

Taehoon Jang orcid: 0000-0002-2123-8427.

Hye Hyeon Kim orcid: 0000-0002-1928-4168.

Kye Hwa Lee orcid: 0000-0002-7593-7020.

Seungsik Hwang orcid: 0000-0002-1558-7831.

Clara Tammy Kim orcid: 0000-0001-7381-7044.

Hoseok Koo orcid: 0000-0001-7856-8083.

Ju Han Kim orcid: 0000-0003-1522-9038.
